# Fine Needle Aspiration Cytology (FNAC) for Chinese Patients With Acral and Cutaneous Melanoma: Accuracy and Safety Analysis From a Single Institution

**DOI:** 10.3389/fonc.2020.01724

**Published:** 2020-10-19

**Authors:** Lingge Yang, Wei Sun, Yu Xu, Xun Zhang, Shengping Wang, Chunmeng Wang, Yong Chen

**Affiliations:** ^1^Department of Musculoskeletal Oncology, Fudan University Shanghai Cancer Center, Shanghai, China; ^2^Department of Oncology, Shanghai Medical College, Fudan University, Shanghai, China; ^3^Department of Ultrasound Diagnosis, Fudan University Shanghai Cancer Center, Shanghai, China; ^4^Department of Diagnostic Radiology, Fudan University Shanghai Cancer Center, Shanghai, China

**Keywords:** fine needle aspiration cytology (FNAC), Chinese patients, acral melanoma, cutaneous melanoma, prognosis

## Abstract

This study aimed to investigate the accuracy and safety of fine-needle aspiration cytology (FNAC) in Chinese patients with acral and cutaneous melanoma, and also to evaluate the influencing factors and their impact on prognosis. Data of 128 patients with stage 0–III acral and cutaneous melanoma treated in Fudan University Shanghai Cancer Center from 2009 to 2016 were collected from a prospective database. Further, 128 patients who did not undergo FNAC but had similar parameters were recruited as the matched group. Clinical features, FNAC status, and recurrence or metastasis status of patients were analyzed for overall survival (OS), melanoma-specific survival (MSS), recurrence-free survival (RFS), and metastasis-free survival (MFS). Of the 128 patients with FNAC, 5.5% (7/128) had a negative cytological diagnosis, 12.2% (5/41) had primary lesions, and 2.3% (2/87) had lesions in lymph nodes. Tumor thickness, status of ulceration, and subtype were not associated with accuracy for both primary and lymph node FNAC. With a median follow-up of 40 months in all patients, 55 had melanoma-specific death; the median OS and MSS were 95 months and 104 months, respectively. Patients with FNAC had significantly worse OS. Tumor progression occurred in 130 patients. The survival analysis revealed differences in OS and disease-free survival between the two groups. FNAC impacted patients' RFS and MFS; the difference in survival curves of RFS and MFS was also statistically significant. FNAC on primary or superficial lymphatic lesions was a good diagnostic tool for Chinese patients with acral and cutaneous melanoma, but it adversely impacted prognosis.

## Introduction

Malignant melanoma (MM) is a malignant tumor caused by excessive proliferation of abnormal melanocytes (1). MM is a highly invasive tumor with a distinct tendency to metastasize; lymphatic and hematogenous metastases occur early in tumor progression. The 5-year overall survival (OS) rate for advanced melanoma and early-stage melanoma is 23 and 98%, respectively (2). Therefore, early diagnosis and screening are important for patients with MM.

In Western countries, patients with cutaneous type as their main type of MM are routinely screened according to the ABCDE rule and undergo an excisional biopsy for small, suspicious lesions (3). However, Chinese people generally have poor health consciousness and insufficient screening. Hence, it is very common that patients would not come to visit unless their primary melanoma or regional lymph nodes become symptomatic or very large. Patients with typical melanoma undergoing a wide local resection have similar subsequences as those with an excisional biopsy in terms of resection and one-stage repair. Hence, the rationality of biopsy, whether excisional or incisional, with histopathology is worth discussing. An incisional biopsy is a traumatic procedure associated with delayed treatment and increased costs. Some scholars tried non-invasive or minimally invasive diagnostic methods, including fine-needle aspiration cytology (FNAC) assessment of primary lesions and clinically enlarged lymph nodes, for further treatment. Preoperative ultrasound could not provide reliable lymph node staging in patients with melanoma (4). FNAC has been applied in clinical practice in many institutions (5–9). However, reports on acral and cutaneous subtypes from China are few. Also, the effect of puncture at primary or lymph node sites on patient prognosis has been rarely reported.

FNAC is an important pathological technique commonly used in patients with suspected cutaneous melanoma on unusual clinical locations and for follow-up in patients with a previous diagnosis of melanoma (8, 10). FNAC has a very limited role in the diagnosis of superficially located melanocytic lesions (8). It is still an important tool to detect suspicious local or regional recurrence in patients with a history of MM; also, it is a reliable diagnostic tool with or without imaging guidance for patients with metastatic MM owing to its high sensitivity (97%) and specificity (99%) (10).

This study analyzed the results of FNAC in 41 patients with melanoma of primary sites and 87 with melanoma of lymph node sites. Further, the accuracy of FNAC in Chinese patients with acral and cutaneous melanoma and influencing factors were evaluated. In addition, the same numbers of patients with acral and cutaneous melanoma without FNAC were matched according to the baseline characteristics to analyze whether FNAC impacted the prognosis of patients.

## Methods

### Patients

A total of 128 patients with stage 0–III MM underwent FNAC followed by surgical treatment in the Department of Musculoskeletal Oncology, Fudan University Shanghai Cancer Center, from November 2006 to June 2018, and were retrospectively examined as the study group. Patients diagnosed with cutaneous or acral melanoma, who underwent no other forms of biopsies before FNAC, were included in the analysis. According to their wishes, these patients underwent FNAC for a preliminary diagnosis to reduce the psychological burden as much as possible during the waiting period for surgery. Among them, 41 patients were on primary sites and 87 on lymph nodes. In addition, the same number of surgically treated patients with acral and cutaneous MM without FNAC from the prospective database were matched and formed the matched group. The matched group was a random selection from all patients in the database and it was matched according to clinical characteristics as much as possible: gender, age, tumor site, tumor size, tumor type, clinical classification and AJCC anatomic stage. A definite diagnosis of MM was confirmed in all patients by pathological diagnosis results after surgery. The medical information of all patients was available, including primary and metastatic tumor sites, results of routine and imaging examinations, treatment records, follow-up data, and disease progression assessed by imaging. The Institutional Review Board approved the study of the cancer center, and all patients were informed and signed relevant documents.

### Fine-Needle Aspiration Cytology

After the physical examination and obtaining the informed consent of each patient, FNAC was performed on suspicious lesions before surgical treatment, according to the patients' wishes, to reduce the psychological burden of the patients. FNAC was performed by a professional cytopathologist at the cancer center, as follows: inquiring about the medical history; determining the location, size, and characteristics of the mass; disinfecting skin; spreading disinfection towels; fixing the mass with the index and middle fingers, puncturing the needle into the mass, using negative pressure for aspiration, and withdrawing the needle after obtaining sufficient specimen. For primary lesions, the most representative site, such as the ulcer or the thickest site of the lesions, was selected for puncture, while for lymph node lesions, the central site of the lymph node was selected for puncture because of the lack of ultrasound guidance. The extracts were smeared, fixed, stained, and then observed under a microscope. Immunohistochemical analysis was further performed on smears of suspected tumor cells with unclear morphology. FNAC positivity was defined as isoform cells with pigmentation and nuclear mitosis in the smear, or combined with immunohistochemistry results indicating malignant tumor cells or suspected malignant tumor cells.

### Treatment

All patients in the study and matched groups were surgically treated with the intent to cure and have full information on primary or regional lymph nodes. For primary lesions, patients with FNAC negativity but clinically suspected to have melanoma underwent an extended resection after a wide local resection of the lesions and pathological diagnosis of melanoma. For patients with FNAC positivity, a sentinel lymph node biopsy was performed after the complete resection of the lesions. Patients with lymph node lesions, who were FNAC negative but pathological positive after lymphadenectomy or FNAC positive, underwent an extended resection of the primary lesions and regional lymph node dissection.

Adjuvant therapies were administered according to the tumor clinical stage and patients' wishes. Based on the *Guidelines of Chinese Society of Clinical Oncology for Melanoma* version 2019, biotherapy was recommended for patients with high-risk melanoma in stages IIB–III. The specific regimen was interferon alfa-2b (IFN-α-2b) 3 MU/m^2^ and interleukin-2 (IL-2) 5 MU/m^2^, intravenous drip, once every other day; the duration of biotherapy was 1 year. Chemotherapy was recommended for patients with confirmed lymph node metastasis, and the regimen was as follows: DTIC (250 mg/m^2^) q.d. by intravenous drip for five consecutive days and DDP (25 mg/m^2^) q.d. for three consecutive days. One course of treatment comprised 4–6 courses for 28 days for each patient.

### Follow-Up and Statistical Analysis

All patients were followed up by telephone and the outpatient service at regular intervals (3- to 6-month intervals postoperatively for 2 years and annually thereafter). The follow-up ended on September 5, 2019. OS was defined as the time from diagnosis to death, and melanoma-specific survival (MSS) as the time from diagnosis to death due to melanoma. DFS was defined as the time from surgical treatment to the events of recurrence, metastasis, death, or the first occurrence of new foci. Recurrence-free survival (RFS) was defined as the time from surgery to the first recurrence of the tumor. MFS was defined as the time from surgery to the first regional or distant metastasis of the tumor. The follow-up data were processed statistically with SPSS 24.0, and the Kaplan–Meier method was used for survival analyses. The log-rank test was used to compare the survival curves of patients in different subgroups. The chi-square test and Student *t*-test were used for comparative analyses between the two subgroups. The differences were considered statistically significant at *P* < 0.05.

## Results

### Characteristics of Patients at Baseline

A total of 128 patients had FNAC, including 69 women (53.9%) and 59 men (46.1.0%), aged 19–85 years, with a median age of 57.5 years. The puncture site was the primary site in 41 patients (32.0%) and the lymph node site in 87 patients (68.0%). The clinical stage was based on the eighth edition of the American Joint Committee on Cancer (AJCC). Forty-one patients (32.0%) were in stage 0, 12 (9.4%) in stage I, 19 (14.8%) in stage II, and 56 (43.8%) in stage III. Among these, 80 cases (62.5%) were of acral type and 48 (37.5%) of cutaneous type. Sixty patients (46.9%) had superficial spreading melanoma, 31 (24.2%) nodular melanoma, 22 (17.2%) lentigo melanoma, and 15 (11.7%) acral lentiginous melanoma.

In addition, the same number of patients with acral or cutaneous melanoma who had not received FNAC from the prospective database were matched based on the baseline characteristics of the 128 patients. No statistically significant difference was found in age, sex, tumor site, tumor size, tumor type, ulcer, clinical classification, Clark level, Breslow thickness, N classification, and AJCC anatomic stage between the two subgroups whether FNAC was performed in primary or lymph node sites. The details are shown in Table 1. No significant difference was found in the baseline characteristics between the FNAC and no-FNAC groups, so the baseline characteristics of patients in the two groups were basically consistent.

**Table 1:** Patient characteristics.

**Variables**	**Primary site**			**Lymph node site**		
	**FNAC**	**No FNAC**	***P*-value**	**FNAC**	**No FNAC**	***P*-value**
Patients		82			174	
Age (years)	64.17 ± 12.84	60.71 ± 9.93	0.1984	53.57 ± 13.53	55.41 ± 12.61	0.3619
**Gender**						
Female	28	28	>0.9999	41	41	>0.9999
Male	13	13		46	46	
**Tumor site**						
Head and neck	6	7	0.9551	5	5	0.8797
Trunk	1	1		11	9	
Extremities	34	33		71	74	
Tumor size (cm)	2.052 ± 1.790	2.578 ± 1.523	0.3413	2.788 ± 1.601	2.688 ± 1.575	0.7707
**Ulceration**						
Yes	10	7	0.4138	15	14	0.8388
No	31	34		72	73	
**Tumor type**						
Acral	26	26	>0.9999	54	52	0.7560
Cutaneous	15	15		33	35	
**Clinical classification**						
Superficial spreading melanoma	16	16	0.9311	44	49	0.4246
Nodular melanoma	12	11		19	11	
Lentigo melanoma	8	7		14	14	
Acral lentiginous melanoma	5	7		10	13	
**Clark level**						
Unknown	23	30		47	54	
I	0	1	0.3467	0	0	0.4618
II	0	0		3	3	
III	1	2		2	2	
IV	11	6		21	22	
V	6	2		14	6	
**Breslow thickness**						
≤ 1 mm	24	29	0.7096	56	63	0.3222
> 1–2 mm	6	4		9	4	
> 2–4 mm	5	4		12	14	
> 4 mm	6	4		10	6	
**N classification**						
N0	29	29	0.8604	43	44	0.9770
N1	5	3		13	11	
N2	4	5		12	12	
N3	3	4		19	20	
**AJCC anatomic stage**						
Stage 0	17	20	0.8168	24	33	0.2428
Stage I	4	4		8	3	
Stage II	8	5		11	8	
Stage III	12	12		44	43	

### Accuracy of FNAC and Its Influencing Factors

In this study, false negativity was found in 7 of the 128 patients in the study group. The false-negative rate was 5.5%. The false-negative rate of primary sites was 12.2% (5/41), higher than that of lymph node sites (2/87, 2.3%). The difference between the two subgroups was not statistically significant (chi-square with Yates' correction *P* = 0.2574). However, the sensitivity of FNAC was higher on lymph node sites than on primary sites. The results of FNAC-positive patients were all positive because only the patients with MM were analyzed in this study. Therefore, the sensitivity and specificity of FNAC was 94.5 and 100%, respectively.

In addition, the patients were divided into false-negative or diagnosis groups based on the results, and the factors affecting the accuracy of FNAC were analyzed. Age (Student *t*-test *P* = 0.2800), sex (chi-square with Yates' correction *P* = 0.8311), tumor site (chi-square test *P* = 0.4444), tumor size (Student *t*-test *P* = 0.4283), ulceration (chi-square with Yates' correction *P* = 0.8964), tumor type (chi-square with Yates' correction *P* = 0.0879), clinical classification (chi-square test *P* = 0.3421), Clark level (chi-square test *P* = 0.3503), Breslow thickness (chi-square test *P* = 0.0978), N classification (chi-square test *P* = 0.4188), AJCC anatomic stage (chi-square test *P* = 0.4461), and ultrasonic examination before FNAC (chi-square with Yates' correction *P* = 0.4369) were not the influencing factors for the accuracy of FNAC. The details are provided in Table 2.

**Table 2:** Relationship between clinicopathological characteristics of patients with melanoma and fine-needle aspiration cytology.

**Variables**	**False-negative**	**Accuracy**	***χ^2^***	***t***	***P*-value**
Cases (%)	7 (5.5%)	121 (94.5%)			
**Puncture site (%)**					
Primary site	5(12.2%)	36 (87.8%)	3.675		0.0553
Lymph node site	2 (2.3%)	85 (97.7%)			
Age (years)	62.57 ± 15.10	56.64 ± 14.00		1.085	0.2800
**Gender**					
Female	3	66	0.0455		0.8311
Male	4	55			
**Tumor site**					
Head and neck	0	11	1.622		0.4444
Trunk	0	12			
Extremities	7	98			
Tumor size (cm)	2.483 ± 2.069	1.921 ± 1.659		0.7953	0.4283
**Ulceration**					
Yes	2	23	0.01696		0.8964
No	5	98			
**Tumor type**					
Acral	7	73	2.912		0.0879
Cutaneous	0	48			
**Clinical classification**					
Superficial spreading melanoma	1	59	3.340		0.3421
Nodular melanoma	3	28			
Lentigo melanoma	2	20			
Acral lentiginous melanoma	1	14			
**Clark level**					
Unknown	1	69			
I	0	0	3.281		0.3503
II	0	3			
III	0	3			
IV	2	30			
V	4	16			
**Breslow thickness**					
≤ 1 mm	2	78	6.301		0.0978
> 1–2 mm	1	14			
> 2–4mm	3	14			
> 4 mm	1	15			
**N classification**					
N0	3	69	2.829		0.4188
N1	2	16			
N2	0	16			
N3	2	20			
**AJCC anatomic stage**					
Stage 0	1	40	2.666		0.4461
Stage I	0	12			
Stage II	2	17			
Stage III	4	52			
**Ultrasound**^$^					
Undone	5	59	0.6045		0.4369
Underwent	2	62			

$ *It is the routine ultrasound examination before surgical resection*.

For the primary lesions, the subtype (acral vs. cutaneous, chi-square with Yates' correction *P* = 0.1878), Breslow thickness (chi-square *P* = 0.7801), ulceration (chi-square with Yates' correction *P* = 0.7552), and tumor size (Student *t*-test *P* = 0.6660) were not associated with accuracy. For the lymph node lesions, the size of lymph nodes (Student *t*-test *P* = 0.5176) and the status of primary lesions were not associated with accuracy. The details are provided in Table 3.

**Table 3:** Relationship between clinicopathological characteristics of patients with melanoma and fine-needle aspiration cytology in the primary or lymph node site.

**Variables**	**Primary site**			**Lymph node site**		
	**False-negative**	**Accuracy**	***P-*value**	**False-negative**	**Accuracy**	***P*-value**
Cases		41			87	
Age (years)	68.80 ± 12.15	63.53 ± 12.55	0.3828	47.00 ± 9.90	53.73 ± 13.61	0.4902
**Gender**						
Female	3	25	0.9302	0	41	0.4959
Male	2	11		2	44	
**Tumor site**						
Head and neck	0	6	0.5564	0	5	0.7940
Trunk	0	1		0	11	
Extremities	5	29		2	69	
Tumor size (cm)	2.380 ± 2.296	2.022 ± 1.646	0.6660	2.500 ± 0.707	1.745 ± 1.628	0.5176
**Ulceration**						
Yes	2	8	0.7552	0	15	>0.9999
No	3	28		2	70	
**Tumor type**						
Acral	5	21	0.1878	2	52	0.5237
Cutaneous	0	15		0	33	
**Clinical classification**						
Superficial spreading melanoma	1	15	0.7922	0	44	0.3238
Nodular melanoma	2	10		1	13	
Lentigo melanoma	1	7		1	18	
Acral lentiginous melanoma	1	4		0	10	
**Clark level**						
Unknown	1	22		0	47	
I	0	0	0.1312	0	0	0.9400
II	0	0		0	3	
III	0	1		0	2	
IV	1	10		1	20	
V	3	3		1	13	
**Breslow thickness**						
≤ 1 mm	2	22	0.7801	0	56	0.1061
> 1–2 mm	1	5		0	9	
> 2–4 mm	2	3		1	11	
> 4 mm	0	6		1	9	
**N classification**						
N0	2	27	0.1033	1	42	0.7216
N1	2	3		0	13	
N2	0	4		0	12	
N3	1	2		1	18	
**AJCC anatomic stage**						
Stage 0	1	16	0.3876	0	24	0.3897
Stage I	0	4		0	8	
Stage II	1	7		1	10	
Stage III	3	9		1	43	
**Ultrasound**						
Undone	5	19	0.1275	0	49	0.1042
Underwent	0	17		2	36	

### Association Between FNAC and Patient Prognosis

**Figure 1:**
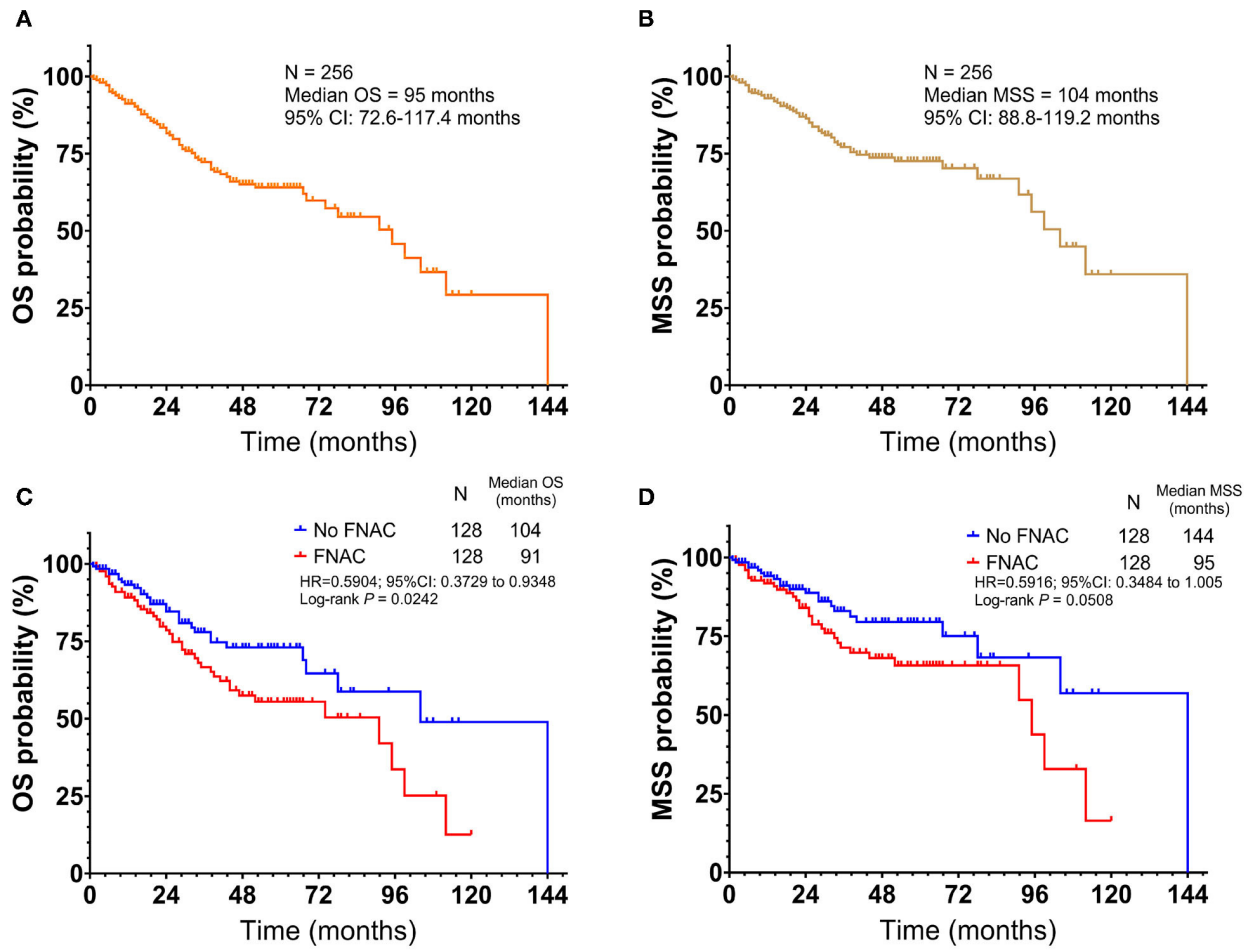
Kaplan-Meier plot of **(A)** overall survival(OS) and **(B)** melanoma-specific survival (MSS) for all patients with acral and cutaneous melanoma. Subgroup analysis by fine-needle aspiration cytology revealed that the no FNAC group exhibited a longer survival than the FNAC group **(C)** in terms of OS (Log-rank test, *P* = 0.0242), **(D)** but a trend was observed for MSS (Log-rank test, *P* = 0.0508). Vertical axis, probability of survival; horizontal axis, survival time in months.

All 256 patients were followed up with a median time of 40 months (1–144 months). Among these, 73 died and 183 survived; the median OS was 95 months [95% confidence interval (CI): 72.6–117.4 months, Figure 1A]. The 1, 3, 5, and 10-year OS rates were 91.2, 72.3, 64.1, and 29.3%, respectively. Further, 44 patients died in the FNAC group and 29 patients in the no-FNAC group. The difference between the two groups was statistically significant (chi-square *P* = 0.0379). Of the patients who died, 55 had melanoma-specific deaths, and the median MSS was 104 months (95% CI: 88.8–119.2 months, Figure 1B). The 1, 3, 5, and 10-year MSS rates were 92.9, 77.1, 72.6, and 35.9%, respectively. Among these, 33 patients in the FNAC group and 22 cases in the no-FNAC group had melanoma-specific deaths, and the difference was not statistically significant (chi-square test *P* = 0.0941). Further analysis found that the differences in the number of patients between the two groups in OS (chi-square test *P* = 0.9438) and MSS (chi-square test *P* = 0.906) of patients with different puncture sites (primary or lymph node site) were not statistically significant. Similarly, the difference on the number of patients between these two groups in the OS (chi-square test *P* = 0.9681) and MSS (chi-square test *P* = 0.7648) of patients with different tumor types (cutaneous or acral) was not statistically significant. However, the survival analysis revealed that patients who did not undergo FNAC had longer survival compared with patients who underwent FNAC, in terms of OS (FNAC vs. no-FNAC = 91 months vs. 104 months, *P* = 0.0242, Figure 1C), but a trend was observed for MSS (FNAC vs. no-FNAC = 95 months vs. 144 months, *P* = 0.0508, Figure 1D).

**Figure 2:**
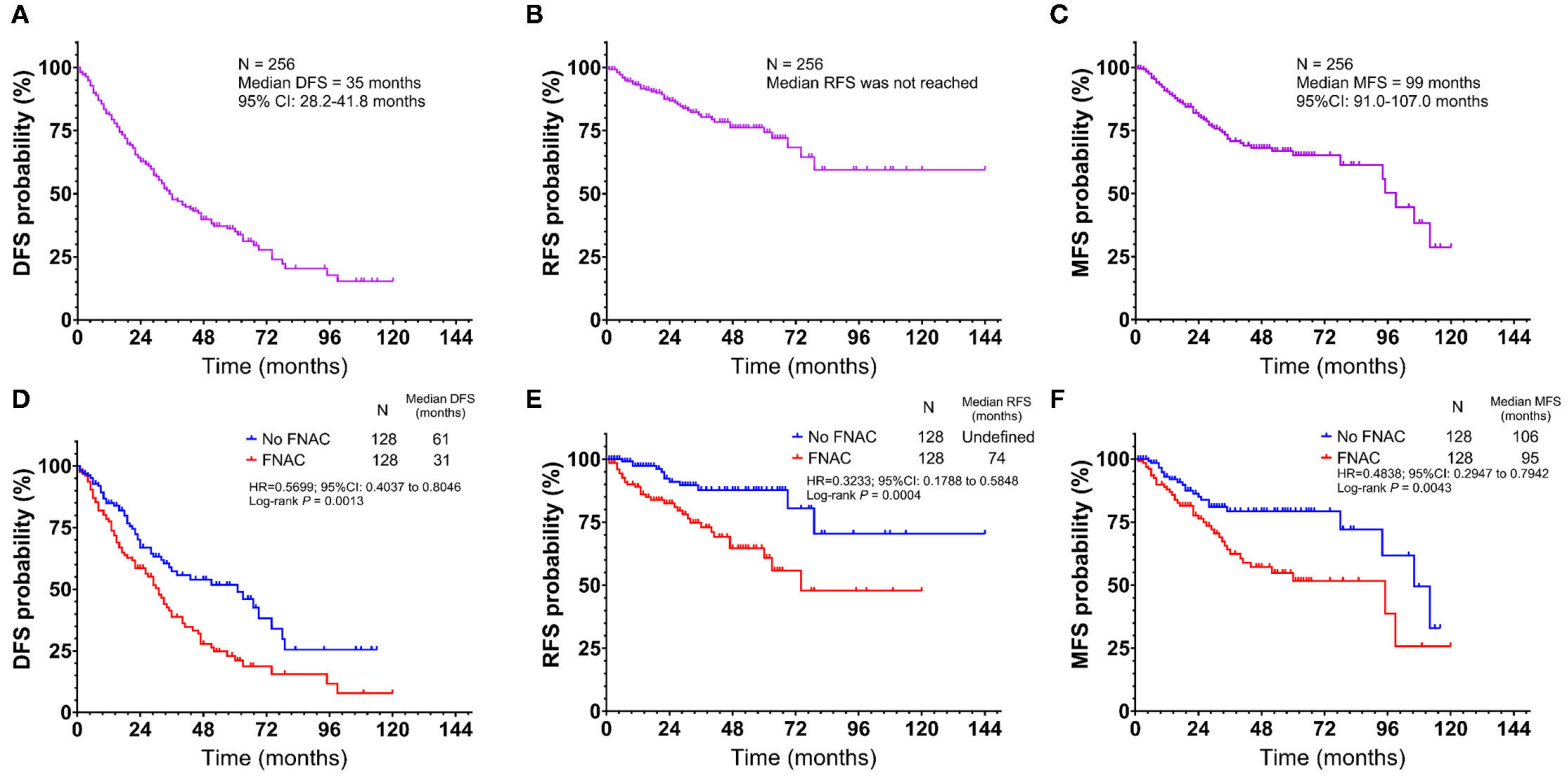
Kaplan-Meier plot of **(A)** disease-free survival (DFS) **(B)** recurrence-free survival (RFS), and **(C)** metastasis-free survival (MFS) for all patients with acral and cutaneous melanoma. Subgroup analysis by fine-needle aspiration cytology revealed that the no FNAC group exhibited a longer survival than the FNAC group in terms of **(D)** DFS (Log-rank test, *P* = 0.0013), **(E)** RFS (Log-rank test, *P* = 0.0004) and **(F)** metastasis-free survival (Log-rank test, *P* = 0.0043). Vertical axis, probability of survival; horizontal axis, survival time in months.

Therefore, the relationship between FNAC and local recurrence, lymph node metastasis, or distant metastasis was further analyzed. Progressive events (recurrence, metastasis, new lesions, or death) occurred in 130 patients, including 78 in the FNAC group and 52 in the no-FNAC group. The difference between these two subgroups was statistically significant (chi-square test *P* = 0.0012). The median DFS was 35 months (95% CI: 28.2–41.8 months, Figure 2A), and the 1, 3, 5, and 10-year DFS rates were 81.3, 47.8, 34.9, and 15.3%, respectively. Among these, 44 had local recurrence (32 in the FNAC group and 12 in the no-FNAC group); the median RFS was not reached (Figure 2B), and the difference between the two subgroups was statistically significant (chi-square test *P* = 0.0009). Further, 63 patients had distant metastasis (40 in the FNAC group and 23 in the no-FNAC group), and the median MFS was 99 months (95% CI: 91.0–107.0 months, Figure 2C), with a statistically significant difference between the groups (chi-square test *P* = 0.0136). In addition, the impact of the puncture site and the tumor type on disease progression was not significant. The difference in DFS was statistically significant (FNAC vs. no-FNAC = 31 months vs. 61 months, *P* = 0.0031, Figure 2D). The RFS and MFS were compared between the FNAC and no-FNAC groups to know whether recurrence or metastasis caused the difference. The main effect of FNAC was on patient's RFS (FNAC vs. no-FNAC = 74 months vs. undefined, *P* = 0.0004, Figure 2E), and the difference in MFS was also statistically significant (FNAC vs. no-FNAC = 95 months vs. 106 months, *P* = 0.0043, Figure 2F). Further analyses showed that the primary puncture did not lead to an increase in the incidence of lymph node metastasis (chi-square test *P* = 0.9565) and distant metastasis (chi-square test *P* = 0.7559). However, a lymph node puncture might cause an increase in the incidence of distant metastasis (chi-square test *P* = 0.0024). Among these, the difference in the increase in lymph node metastasis was statistically significant (chi-square test *P* = 0.0019), but no significant difference was observed in the increase in distant organ metastasis (chi-square test *P* = 0.2267). The details are provided in Table 4.

**Table 4:** Relationship between fine-needle aspiration cytology (FNAC) and patient prognosis.

**Variables**	**FNAC**	**No FNAC**	***χ^2^***	***P*-value**
Cases	128	128		
**Overall survival**						
Alive	84	99	4.312	0.0379^*^
Death	44	29		
Primary site	14	9	0.0050	0.9438
Lymph node	30	20		
Acral	26	17	0.0016	0.9681
Cutaneous	18	12		
**Melanoma specific death**						
No	95	106	2.802	0.0941
Yes	33	22		
Primary site	11	7	0.0138	0.9066
Lymph node	22	15		
Acral	22	17	0.0895	0.7648
Cutaneous	11	10		
**Disease progression**						
No	50	76	10.57	0.0012^**^
Yes	78	52		
Primary site	23	16	0.0244	0.8758
Lymph node	55	36		
Acral	45	32	0.1911	0.6620
Cutaneous	33	20		
**Local recurrence**						
No	96	116	10.98	0.0009^***^
Yes	32	12		
Primary site	10	4	0.0046	0.9462
Lymph node	21	8		
Acral	20	6	0.5641	0.4526
Cutaneous	12	6		
**Distant metastasis**						
No	88	105	6.085	0.0136^*^
Yes	40	23		
Primary site (*n* = 41 in each group)	11	11	0	>0.9999
Without metastasis	30	30		
Lymph node metastasis	7	6	0.0030	0.9565
Other metastasis	4	5	0.0966	0.7559
Lymph node (*n* = 87 in each group)	29	12	9.222	0.0024^**^
Without metastasis	58	75		
Lymph node metastasis	20	6	9.658	0.0019^**^
Other metastasis	9	6	1.462	0.2267
Acral	23	12	0.1678	0.6821
Cutaneous	17	11		

* *P < 0.05*,

** *P < 0.01*,

*** *P < 0.001*.

## Discussion

Patients with melanoma have the characteristics of thicker infiltration depth, higher ulcer incidence, and higher lymphatic metastasis rate in China compared with Western countries. Therefore, the prognosis of Chinese patients with melanoma is relatively poor (11). Similarly to solid tumors, the earlier the clinical stage, the better the patient's prognosis. According to statistics, the 5 and 10-year MSS rates of patients in stages I, II, and III were 98% and 95%, 90% and 84%, and 77% and 69%, respectively (12). Therefore, early diagnosis is particularly important.

FNAC is a convenient, fast, and economical pathological technique for diagnosis. It provides an effective and accurate assessment of suspicious nodules in patients diagnosed with malignant tumors (6, 7, 13). In the case of MM, FNAC was used to detect tumor cells in the swollen lymph nodes of patients with or without ultrasound guidance, with high sensitivity and specificity (5, 6, 9). In addition, FNAC, as the first workup of stage III patients with clinically or pathologically detectable intralymphatic metastases, has the characteristics of low cost and low risk (5–9, 13). In the present study, FNAC had a highly accurate diagnosis. The overall diagnostic rate was 94.5%; the diagnostic rate of primary puncture was 87.8%, slightly lower than 97.7% for a lymph node puncture.

The diagnostic rate of primary puncture was not as high as that of lymph node puncture. The most important reason for this might be that the majority of patients (80/128, 62.5%) who underwent FNAC had melanoma *in situ*. The tumors in these patients were thin, or the extent of lymph node lesions was small. The tissues aspirated by FNAC under non-imaging guidance were mostly normal tissues, and the aspiration of tumor cells was difficult. Meanwhile, the morphology of melanoma cells was diverse, and the typical morphology was destroyed or masked by necrotic tissues (8, 9). Therefore, the five FNAC-negative specimens in the primary site were not good enough for diagnosis. Moreover, many studies suggested that insufficient specimens might be an important cause of FNAC-negative results (10, 13–16). No accurate records were available in the present study, in terms of the amount of specimens aspirated by FNAC, for further analysis in the two groups.

The statistical analysis in this study found that the Breslow thickness (chi-square test *P* = 0.0978) and the tumor type (chi-square with Yates' correction *P* = 0.0879) were not the main factors affecting the misdiagnosis of FNAC. However, these two characteristic parameters showed a certain trend between the groups because the *P*-values were close to 0.05. This was probably because of the small sample size in the false-negative group. Therefore, in clinical practice, FNAC increased the risk of misdiagnosis for patients with suspected acral and cutaneous melanoma, and hence FNAC could not be recommended for these patients in China. A complete biopsy is recommended for MM originating from the skin or extremities to measure size and thickness of melanoma and facilitate diagnosis, thus obtaining an R0 margin to reduce the recurrence rate and benefit patient prognosis (17, 18). Even if the primary sites of the lesions were large or the metastasis sites needed to be confirmed, a local excisional biopsy should be performed to obtain a correct diagnosis and accurate clinical stage.

This study analyzed the difference in survival between 128 patients who underwent FNAC and 128 patients who did not undergo FNAC in the prospective database. The differences in OS, DFS, RFS, and MFS were not statistically significant between the two subgroups. Interestingly, the comparison of the total deaths in the two groups showed that 18 patients did not die from MM, with a statistically significant difference in OS (*P* = 0.0379), but no statistically significant difference in MSS (*P* = 0.0941) between the two groups. The baseline characteristics of 18 patients were analyzed, revealing that all of them were older than 65 years. These patients might be suffering from other underlying diseases or financial crisis, and their attitude toward treatment was not very positive.

The results suggested that FNAC did not prolong the survival time of Chinese patients with stage 0–III acral and cutaneous melanoma. It was presumed that FNAC might result in tumor seeding in the tract. Many epithelial-derived tumors showed that a biopsy could lead to the needle-track implantation of metastases, such as gastrointestinal (19, 20), pancreas (21), thoracic (22, 23), thyroid (24), and urological malignancies (25). In terms of melanoma, a case report showed tumor seeding in a 92-year-old male patient with MM of the head and neck region due to FNAC on a lymph node, suggesting that patients undergoing FNAC should also undergo a definitive surgery involving the FNA tract so as to reduce the incidence of complications, such as the dissemination of malignant cells (26). Some reports also showed that the incidence of tumor seeding on the FNAC tract was higher in ocular melanomas (27–29). One of these case reports confirmed a local melanoma growth in the puncture area (29). Therefore, melanoma dissemination and metastasis through the FNAC tract was possible, indicating that patients with MM undergoing FNAC were more likely to have local recurrence, resulting in poor prognosis. However, this hypothesis was not proved in the present study because no related pathological examinations were performed. In addition, the inflammatory response at the puncture sites also led to the proliferation of melanoma cells. Hanahan et al. (30) proposed that one of the 10 characteristics of tumors was the tumor-promoting inflammatory response. Inflammatory cells can induce and help maintain tumor angiogenesis by releasing inflammatory factors, stimulating tumor cell proliferation and thus supporting their metastasis and spread and promoting tumor tissue infiltration. Inflammatory biomarkers were found to be associated with the prognosis of patients with melanoma (31–34). FNAC is mainly used as a diagnostic tool and cannot completely remove tumors. Hence, tumor cells may form micrometastases before a patient undergoes therapeutic surgery, or undergoes hematogenous metastasis or lymphatic metastasis on exposure to inflammatory factors released by immunity inflammatory cells. Therefore, it is possible that the prognosis of patients with FNAC may be poorer than that of patients without FNAC.

This study had several limitations. First, the patients were enrolled from a single center in China and were all Asians. Hence, the results were not directly representative of melanoma characteristics of other races. The results of this study indicated that FNAC was generally not recommended for patients with acral and cutaneous melanoma in China, whether in primary or lymph node sites. After obtaining the informed consent of patients, the primary lesion or the site suspected of having lymph node metastasis should be removed as completely as possible. Second, the study did not investigate whether the primary puncture had an effect on the positive rate of sentinel lymph node biopsy (SLNB), which is a standardized method for assessing regional lymph node metastasis by pathological staging. SLNB helps obtain N staging of patients accurately and provides a clinical basis for follow-up treatment. It can improve patient RFS, even if a significant impact on OS is lacking (35). All of the 41 patients with primary melanoma underwent FNAC, and their matched patients were not SLNB positive; therefore, the issue regarding the effect of puncture on SLNB positivity could not be discussed. In addition, the false-negative rate was used to represent the accuracy of FNAC in the present study, which was not accurate. However, all patients in the database had melanoma; therefore, the patients with FNAC-positive included in the study were all positive postoperative pathologic results. In other words, the false-positive rate was zero. Therefore, the false-negative rate was the best indicator of the accuracy of FNAC results in the present study. In addition, the baseline characteristics of patients affecting the accuracy of FNAC were not found in this study. The baseline characteristics that can affect false-negative FNAC results include difficult aspiration sites (such as deep inguinal lymph nodes), superficial subcutaneous lesions associated with fibrosis or previous scars, and so forth (16). In addition, palpation-guided FNAC could increase the probability of false-negative results compared with image-guided FNAC (14). However, patients in this study did not undergo image-guided FNAC. The aspiration sites were superficial lymph nodes. No other forms of biopsies were performed before the patients underwent FNAC, and no superficial subcutaneous lesions were found. Therefore, these factors could not be analyzed. Finally, the aim of this study was to investigate the application of FNAC in patients with MM originating from the skin or extremities in China. Hence, the effect of FNAC on the prognosis of patients was mainly analyzed. The stratification factors were the puncture site and the tumor type; the other factors influencing the prognosis of patients were not analyzed. However, patient-specific factors such as age and sex, and tumor-specific factors such as Breslow thickness, ulcer, and mitotic rate were independent predictive parameters affecting the prognosis of patients (3, 12, 36, 37). The patients in this study were not specific for these factors, and therefore these factors were not fully discussed.

## Conclusions

In summary, FNAC is a very important procedure for clinical decision-making and management of patients with MM in Western countries because of its high sensitivity and specificity, despite its potential complications of tumor seeding. However, FNAC on both primary and lymph node sites increases the risk of adversely affecting the prognosis for Chinese patients with acral and cutaneous melanoma. In the case of patients with non-stage IV cutaneous or acral MM, lesions are superficial and their complete resection is easy. Therefore, complete resection is recommended instead of FNAC.

## Data Availability Statement

The raw data supporting the conclusions of this article will be made available by the authors, without undue reservation.

## Ethics Statement

The studies involving human participants were reviewed and approved by the Ethics Committee of Fudan University Shanghai Cancer Center. The patients/participants provided their written informed consent to participate in this study.

## Author Contributions

YC and CW: conceptualization and supervision. LY, WS, and YX: data curation and formal analysis. LY, XZ, SW, YC, and CW: methodology. LY, SW, YX, and CW: software. LY, WS, XZ, YC, and CW: validation. LY, YX, XZ, SW, and YC: visualization. LY: writing - original draft. YC and CW: writing - review and editing. All authors read and approved the final manuscript.

## Conflict of Interest

The authors declare that the research was conducted in the absence of any commercial or financial relationships that could be construed as a potential conflict of interest.
